# Ozonated *Aloe vera* Oil Effective Increased the Number of Fibroblasts and Collagen Thickening in the Healing Response of Full-Thickness Skin Defects

**DOI:** 10.1155/2021/6654343

**Published:** 2021-02-09

**Authors:** Ahsanu Taqwim Hidayat, Muhamad Thohar Arifin, Muhammad Nur, Muflihatul Muniroh, Neni Susilaningsih

**Affiliations:** ^1^Master Student of Biomedical Sciences, Faculty of Medicine, Diponegoro University, Semarang, Indonesia; ^2^Department of Neurosurgery, Faculty of Medicine, Diponegoro University, Semarang, Indonesia; ^3^Division of Physics, Faculty of Science and Mathematics, Diponegoro University, Semarang, Indonesia; ^4^Department of Physiology, Faculty of Medicine, Diponegoro University, Semarang, Indonesia; ^5^Department of Histology, Faculty of Medicine, Diponegoro University, Semarang, Indonesia

## Abstract

**Objective:**

This study aimed to examine the effectiveness of ozonated *Aloe vera* oil on the wound healing response of full-thickness defect tissue in Sprague-Dawley rats, assessed by collagen thickness and the number of fibroblasts.

**Methods:**

This was an experimental research method using control groups and treatment groups with a posttest only control group design. The results showed that collagen thickness in wounds tended to increase, assessed on day 3 and day 7 using Masson's trichrome staining and microscopic evaluation.

**Results:**

There was a significant difference in the number of fibroblasts between the two control and treatment groups on days 3 and 7 tested using one-way Kruskal–Wallis test, with a value of *p*=0.001(*p* < 0.05), resulting in a significant difference in wound size reduction between the groups. Further post hoc analysis using the Mann–Whitney test indicated a significant difference between the control groups and the treatment groups (P0, P1 versus P3, P4, P5, P8, P9, and P10) with a value of *p*=0.009(*p* < 0.05).

**Conclusions:**

Ozonated *Aloe vera* oil is effective in increasing the healing response of full-thickness defects, leading to the increase in the number of fibroblasts and collagen thickening that in turn accelerates wound healing in Sprague-Dawley rats.

## 1. Introduction

Full-thickness wound is an injury that extends from the epidermis, dermis, fat layer, fascia, even to the bone [1]. Fibroblast proliferation occurs from day 4 to day 21 after the trauma. In this phase, the platelet-rich-fibrin matrix and macrophages are gradually replaced by granulation tissue, which is composed of clusters of fibroblasts, macrophages, and endothelial cells that form the extracellular matrix and neovascularization. During the fibroplasia phase, wounds are filled by inflammatory cells, fibroblasts, and collagen, forming reddish tissues with bumpy surfaces called granulation tissue. Collagen is produced by fibroblasts during the proliferative phase and contributes to remodeling and linking wounds, allowing the skin to have tensile strength, as well as to act as a framework and scaffold for the movement of fibroblasts and other cells in the wound healing process. Fibroblasts help break down fibrin clots, form a new extracellular matrix (ECM), and are associated with the formation of collagen structures [2]. One of the primary functions of fibroblasts and myofibroblasts is to facilitate greater wound size reduction by providing a contractile force that brings together two edges of the wound [3].

*Aloe vera* has been proven to improve wound healing by modulating inflammation, providing greater wound size reduction, and improving wound epithelialization. *Aloe vera* can also improve wound healing by promoting proliferation and migration of fibroblasts and keratinocytes [4]. Lesions treated with *Aloe vera* have better biochemical, morphological, and biomechanical properties of the wound, i.e., increasing maximum load, ultimate strength, and modulus of elasticity. Ozone therapy is an alternative therapy that can be used as a disinfectant and can induce strong oxidative stress, thereby stimulating the protective mechanisms of cells and organs. Anastomotic healing using topical ozone administration has the same effectiveness as a treatment with hyaluronic gel, but is more effective in preventing hyperpigmented lesions. Ozone therapy inactivates bacteria by damaging bacterial cell envelopes through oxidized phospholipids and lipoproteins, inhibiting fungal growth, damaging viral capsids, and interfering the bacterial reproductive cycle [5]. Ozone therapy can be used as an adjuvant or an alternative to the standard therapy in patients with various types of injuries [5].

This study reports the effect of ozone administration using ozonated *Aloe vera* oil in accelerating the healing time of full-thickness defects in Sprague-Dawley rats assessed by the number of fibroblasts and collagen thickness.

## 2. Methods

This study is an experimental study with a posttest only randomized control group design to examine the effect of *Aloe vera* on fibroblasts and collagen thickness. The animals used in this study were male Sprague-Dawley rats, with the following inclusion criteria: male Sprague-Dawley rats aged 2 months, having healthy status (active movement), weighing 250 ± 50 grams, and showing no observable abnormalities. The rats were acclimatized in the laboratory for one week in individual cages given with 12 hours of light and 12 hours of darkness. During the study, the samples were maintained at room temperature, exposed to light for 12 hours, and were fed sufficiently. Ozonated *aloe vera* was obtained from the Plasma Research Laboratory of Diponegoro University, Indonesia, which is derived from a mixture of concentrated ozone and *aloe vera* oil. The samples of the ozonated oil were taken to be tested using a spectrometer or dissolved ozone meter kit. The ozone level in the ozonated oil was measured by titration with potassium iodide.

### 2.1. Procedures

#### 2.1.1. Wound Incision

The rats were incised on the back with a diameter of 1 centimeter as a model for full-thickness defect. For wound incision of full-thickness defect, the rats were anesthetized with ketamine-xylazine anesthetic mixture (ketamine 80 mg/kg BW; xylazine 10 mg/kg BW) intraperitoneal injection. The rats were placed in the left lateral decubitus position. The round wound was made on the back of the rats, with a diameter of 1 centimeter. The wound was created at the base of the panniculus carnosus.

#### 2.1.2. Production of Ozonated *Aloe vera*

The manufacture and testing of ozonated *Aloe vera* were carried out at the Plasma Research Center (PRC), Diponegoro University, Indonesia. *Aloe vera* oil was obtained by extracting *Aloe vera* leaves with 250 ml aceton for 24 hours in Soxhlet apparatus. The solution was then concentrated by rotary evaporator at 40^o^C and room temperature afterward. The tools used for making ozonated oil were ozone generators (ozone generator manufactured in house by Plasma Research Center, Diponegoro University) and magnetic stirrers. The ozone outlet is connected to an antioxidation hose with a diffuser which served to increase the effectiveness of ozone absorption in the oil. Magnetic stirrers were used to facilitate the ozone dissolving process into the oil. The oil used in this study was *Aloe vera* extract. Ozone was dissolved into *Aloe vera* with a volume of 40 cc in each cycle and an oxygen flow rate of 1.5 liters/minute with an ozone concentration of 3360 ppm. The duration of the ozonization process varied depending on the concentration of ozonated oil from 30 to 60 to 120 minutes. In this experiment, the ozonated oil concentration was 600 mg/ml, 1200 mg/ml, and 1800 mg/ml. The ozonated oil had undergone the peroxide value (PV) evaluation.  Table 1 shows the PV concentration in every ozone concentration dose used in this experiment.

#### 2.1.3. Experimental Procedures

*Aloe vera*, gentamicin, and ozonated *Aloe vera* oil were applied to the wound once a day, covering the entire wound surface for each treatment.

### 2.2. Treatment Groups

- The positive control group (P1 and P6): in the full-thickness defect healed group, nonozonated *Aloe vera* oil was administered to the rats
- The positive control group (P2 and P7): gentamicin was administered to treatment group 1 as a standard therapy
- Group P3: in treatment group 1, the subjects received the ozonated *Aloe vera* oil therapy that contained 600 mg of ozone, terminated on day 3
- Group P4: in treatment group 2, the subjects received the ozonated *Aloe vera* oil therapy that contained 1200 mg of ozone, terminated on day 3
- Group P5: in treatment group 3, the subjects received the ozonated *Aloe vera* oil therapy that contained 1800 mg of ozone, terminated on day 3
- Group P8: in treatment group 1, the subjects received the ozonated *Aloe vera* oil therapy that contained 600 mg of ozone, terminated on day 7
- Group P9: in treatment group 2, the subjects received the ozonated *Aloe vera* oil therapy that contained 1200 mg of ozone, terminated on day 7
- Group P10: in treatment group 3, the subjects received the ozonated *Aloe vera* oil therapy that contained 1800 mg of ozone, terminated on day 7

The treatments for the rats were terminated according to the study design on day 3 and day 7 in each group. Wound tissue is removed by excising the largest part of the wound including normal skin tissue. The excised tissue was immediately spread out on the paper to make it easier to cut using a microtome. Afterward, it was fixed in 10% buffered formalin and embedded in paraffin blocks and then cut to a thickness of 4–6 microns for histopathological examination with H&E staining. Fibroblast counting was performed by two independent examiners based on cell morphology using Optilab Pro (Miconos, Indonesia). Staining and examination of collagen and fibroblasts were conducted at the Laboratory of Anatomical Pathology, Faculty of Medicine, Universitas Sebelas Maret, Solo.

### 2.3. Statistical Analysis

Data analysis was performed using computer software. This study used the Kruskal–Wallis test to determine whether there is a significant difference in the score of more than one group of variables, by comparing the data from the treatment groups. Normality tests of the data were conducted using the Shapiro–Wilk test.

Due to nonnormal distribution of the data, the nonparametric test of Kruskal–Wallis test was used. The *p* value is considered significant at *p* < 0.05. If the statistical test indicates failure in rejecting H_0_ (no significant difference between the groups), the post hoc analysis will not be conducted. Meanwhile, in case a significant difference is found, the Mann–Whitney test is performed. The Mann–Whitney test is conducted if a significant difference in variance is observed (significance at *p* < 0.05).

### 2.4. Ethical Approval

This study has gained ethical approval from the Health Research Ethics Committee of the Faculty of Medicine, Diponegoro University, no. 132/EC/H/KEPK/FK-UNDIP/X/2019. All experimental animals were managed and cared for in compliance with the animal welfare standards.

## 3. Results

The result of observation on fibroblasts is presented in Tables 2–4. From the Kruskal–Wallis test followed by the post hoc analysis using the Mann–Whitney test, it was observed that the number of fibroblasts in full-thickness skin defects tended to increase in wound care using 1800 mg ozonated *Aloe vera oil* compared to wound care receiving only *Aloe vera* oil (K1+ and P3) on day 3 by 110.6 (39.2–157.2) *µ*m^2^ and day 7 by 242.2 (168.8–265) *µ*m^2^. Administration of ozonated *Aloe vera* oil to each group of Sprague-Dawley rats with full-thickness defect tissue has been found to enhance the number of fibroblasts. Apparently, administering certain doses of ozonated *Aloe vera* oil (1200 mg and 1800 mg) in the treatment groups on day 3 (P4 to P5) and the treatment group on day 7 (P9 to P10) significantly led to the higher number of fibroblasts in the wound healing process, compared to administration of ozonated *Aloe vera* oil at a dose of 600 mg (P3 and P8), with 110.6 (38–116.8) *µ*m^2^, 110.6 (39.2–157.2) *µ*m^2^, on day 7 with 169.2 (97–204.8) *µ*m^2^ and 242.2 (168.8–265) *µ*m^2^, compared 106.6 (57–117) *µ*m^2^ to 141.6 (78.2–208.6) *µ*m^2^. The number of fibroblasts was likely to rise in the wound healing process using ozonated *Aloe vera* oil compared to wound healing using gentamicin (K2+). Nevertheless, there was no significant difference between P4 and P5. This shows that the administration of ozonated *Aloe vera* oil has a significant effect in increasing the wound healing response to full-thickness defect, which causes an increase in the number of fibroblasts in the Sprague-Dawley rats.

### 3.1. Description of Visual Observation on Fibroblasts

**Figure 1:**
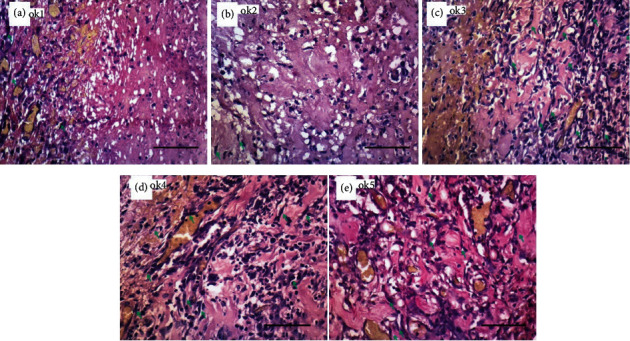
The number of fibroblasts on day 3. (a) Control group with nonozonated *Aloe vera* oil. (b) Control group with gentamicin application. (c) Treatment group with 600 mg ozonated *Aloe vera* oil. (d) Treatment group with 1,200 mg ozonated *Aloe vera* oil. (e) Treatment group with 1,800 mg ozonated *Aloe vera* oil. Scale bar 50 *μ*m.

It was observed that the number of fibroblasts seen on day 3 varied according to each of the groups of P1 (the control group with *Aloe vera* oil), P2 (the control group with gentamicin), P3 (the treatment group given ozonated *Aloe vera* oil at a dose of 600 mg), P4 (the treatment group given ozonated *Aloe vera* oil at a dose of 1200 mg), and P5 (the treatment group given ozonated *Aloe vera* oil at a dose of 1800 mg) (Figure 1).

**Figure 2:**
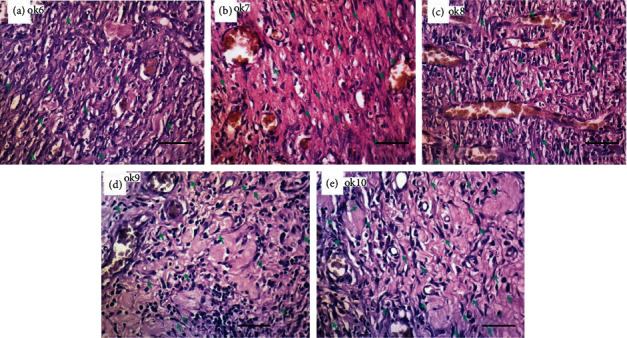
The number of fibroblasts on day 7. (a) Control group with nonozonated *Aloe vera* oil. (b) Control group with gentamicin application. (c) Treatment group with 600 mg ozonated *Aloe vera* oil. (d) Treatment group with 1,200 mg ozonated *Aloe vera* oil. (e) Treatment group with 1,800 mg ozonated *Aloe vera* oil. Scale bar 50 *μ*m.

Meanwhile, the number of fibroblasts on day 7 in each of the groups of P6 (the control group with *Aloe vera* oil), P7 (the control group with gentamicin), P8 (treatment group with ozonated *Aloe vera* oil at a dose of 600 mg), P9 (treatment group with ozonated *Aloe vera* oil at a dose of 1200 mg), and P10 (the treatment group with ozonated *Aloe vera* oil at a dose 1800 mg) increased compared to day 3 of ozonated *Aloe vera* administration (Figure 2).

From the two pictures above, it can be seen that the number of fibroblasts rose in P4 and P5 on day 3 and P9 and P10 on day 7, showing the increase in the number of fibroblasts during the wound healing.

### 3.2. Collagen Thickness

The normality test is the first prerequisite statistical test for the parametric test. The results of the normality test are shown in Tables 5 and 6. The results of the Kruskal-Wallis indicated a value of 0.115 (*p* > 0.05) showing no significant difference between at least two of the treatment groups.

**Figure 3:**
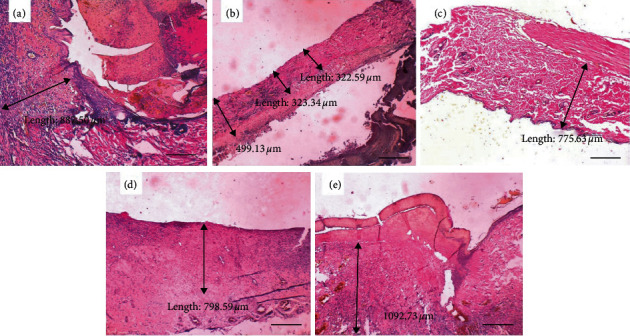
The thickness of collagen on day 3. (a) Control group with nonozonated *Aloe vera* oil. (b) Control group with gentamicin application. (c) Treatment group with 600 mg ozonated *Aloe vera* oil. (d) Treatment group with 1,200 mg ozonated *Aloe vera* oil. (e) Treatment group with 1,800 mg ozonated *Aloe vera* oil. Scale bar 100 *μ*m.

Figure 3 illustrates the collagen thickness on day 3 in each of the groups of P1 (the control group with *aloe vera* oil), P2 (the control group with gentamicin), P3 (the treatment group with ozonated *Aloe vera* oil dose at a dose of 600 mg), P4 (the treatment group with ozonated *Aloe vera* oil at a dose of 1200 mg), and P5 (the treatment with ozonated *Aloe vera* oil at a dose of 1800 mg). It was observed that collagen thickness improved due to the effect of ozonated *Aloe vera* oil (Figure 3).

**Figure 4:**
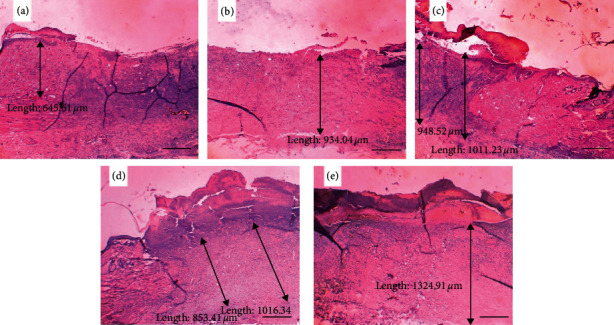
The thickness of collagen on day 3. (a) Control group with nonozonated *Aloe vera* oil. (b) Control group with gentamicin application. (c) Treatment group with 600 mg ozonated *Aloe vera* oil. (d) Treatment group with 1,200 mg ozonated *Aloe vera* oil. (e) Treatment group with 1,800 mg ozonated *Aloe vera* oil. Scale bar 100 *μ*m.

Meanwhile, in Figure 4, the collagen thickness on day 7 was divided according to each of the groups of P6 (the control group with *Aloe vera* oil), P7 (the control group with gentamicin), P8 (the treatment group with ozonated *Aloe vera* oil at a dose of 600 mg), P9 (the treatment group with ozonated *Aloe vera* oil at a dose of 1200 mg), and P10 (the treatment group with ozonated *Aloe vera* oil at a dose of 1800 mg). There was an increase in collagen thickness due to the effect of ozonated *Aloe vera* oil (Figure 4).

## 4. Discussion

Administration of ozonated *Aloe vera* oil has an effect in improving the wound healing response to full-thickness defects, leading to the increase in the number of fibroblasts and collagen thickness that in turn accelerates wound healing in the Sprague-Dawley rats. This also supports our previous finding as the ozonated *Aloe vera* oil increased macrophage counts and epithelization process [6].

The results of this study indicated that collagen in the full-thickness defect treated with ozonated *Aloe vera* oil is thicker than that treated with *Aloe vera* oil and gentamicin ointment. Ozonated *Aloe vera* oil increases reactive oxygen species (ROS) and reactive nitrogen species (RNS) around the wound site, such as platelets, macrophages, fibroblasts, endothelial cells, and keratinocytes that act as wound healing radicals [7]. Fibroblasts around the wound site will produce collagen, which is an undifferentiated mesenchymal cell. Fibroblasts will produce mucopolysaccharides, proline, and aminoglycine acid, which serve as the basic ingredients of collagen fibers that grip the wound edges [8].

Fibroblasts are the primary cells in the proliferative phase that play a central role in providing the extracellular matrix as a framework for keratinocyte migration [9]. The higher number of fibroblasts in the treatment groups leads to faster epithelialization in the treatment group than the positive control wound group as seen in Figures 1 and 2. The statistical analysis was conducted to compare the wound size in the control groups and the treatment groups, revealing the increase in the number of fibroblasts in the treatment groups.

During the proliferative phase, the wound is filled with inflammatory cells, fibroblasts, and collagen, which form reddish tissue with bumpy surfaces called granulation tissue. Subsequently, the epithelium in the wound edge consisting of basal cells detaches from the base and moves outward to fill the wound surface. The site is then filled with new cells formed by the mitosis process. The migration process only occurs in a lower or flat direction. This process only stops after the epithelium connects each other and covers the wound surface. Once the wound surface is closed, the fibroplasia phase of the formation of granulation tissue will also stop and the maturation process begins in the remodeling phase.

Collagen on days 3 and 7 of observation was thicker in the treatment groups than the positive control group. In normal tissues, the secretion and activity of matrix metalloproteinase-1 (MMP-1) is very low, but in injured or inflamed tissues the production and secretion of MMP-1 will rise. This is confirmed by the results of the study where collagen was thicker in the treatment groups on days 3 and 7 of observation because ozonated *Aloe vera* inhibits MMP-1 production so that collagen synthesis increased.

The effect of *Aloe vera* on wound healing was assessed by collagen thickness and the number of fibroblasts in the Sprague-Dawley rats. Collagen thickness was calculated on days 3 and 7 using Van Gieson and assessed microscopically in mm and photographed using Optilab pro (Miconos, Indonesia). Meanwhile, calculation of the number of fibroblasts was performed on days 3 and 7 after the treatment, assessed using Van Gieson's stain and microscopically assessed at 400x magnification with square micrometer area (*µ*m^2^).

The number of fibroblasts in the full-thickness defect treated with ozonated *Aloe vera* oil was higher than that treated with *Aloe vera* oil only and gentamicin ointment. Several previous studies have shown that ozone plays an important role in wound healing and antimicrobial activity [10]. Ozonated ointment increases reactive oxygen species (ROS) at the wound site acting as a secondary messenger for various immunocytes and nonlymphoid cells involved in the wound repair process and plays a pivotal role in coordinating the recruitment of lymphoid cells to the wound site and effective tissue repair. ROS are also able to regulate blood vessel formation (angiogenesis) which optimizes blood perfusion to the wound healing area [11]. With regard to immunity, ROS acts through phagocytes which induce the emergence of large numbers of ROS killing pathogens in the wound, which results in bacterial damage. The induction of large amounts of ROS as discussed above refers to ROS burst, which is preceded by a respiratory burst with a considerable amount of oxygen uptake. Respiratory burst causes the release of ROS and RNS in large numbers, which kill pathogens phagocytosed by these cells. In wound healing, cells such as platelets, macrophages, fibroblasts, endothelial cells, and keratinocytes use ROS and RNS as radicals in wound healing [11]. Therefore, ROS can be used to heal chronic wounds that are difficult to treat [11].

The limitation of this study involves the diversity of hygiene of each rat, where this affects the wound healing process. Furthermore, the phase of wound healing that occurred on days 3 and 7 of the sampling cannot be determined due to the plausible difference in the wound healing process between the Sprague-Dawley rats and humans. Further studies including in vitro assays are required to see whether ozonated *Aloe vera* treatment has a direct impact on fibroblasts proliferation to support our finding.

## 5. Conclusions

The administration of ozonated *Aloe vera* oil at a dose of 1800 mg ozone significantly increases the number of fibroblasts in the full-thickness defect, with which the group that received such dose has the significantly higher number of fibroblasts than the control group that was given only *Aloe vera* oil and gentamicin and there was no significant difference when compared to the low-dose ozonated *Aloe vera* oil group. The administration of ozonated *Aloe vera* oil significantly improves collagen thickness in full-thickness defect wounds in the treatment group compared to the control group that was only given *Aloe vera* oil and gentamicin, and there was no significant difference in collagen thickness between the group given high dose ozonated *Aloe vera* and the group with low dose.

## Figures and Tables

**Table 1:** Results of calculation of the peroxide value based on the dose of ozonated *Aloe vera* oil.

No.	Dose of ozonated *Aloe vera* oil (mg/ml)	Peroxide value milliequivalents per 1000 grams
1	0	66.88 ± 1.71
2	600	654.01 ± 6.44
3	1200	1140.93 ± 6.06
4	1800	1772.81 ± 14.66

**Table 2:** Descriptive statistics and normality testing of fibroblast data using the Shapiro–Wilk test.

Group	Mean ± SD	Median (min-max)	*p*	Transf.
P1	78.64 ± 24.76	69.2 (53–108.2)	0.308^*∗*^	0.424^*∗*^
P2	70.52 ± 19.13	64 (54.6–99.4)	0.293^*∗*^	0.371^*∗*^
P3	99.12 ± 23.97	106.6 (57–117)	0.018	0.006
P4	97.44 ± 33.34	110.6 (38–116.8)	0.002	0.001
P5	97.12 ± 48.23	110.6 (39.2–157.2)	0.710^*∗*^	0.521^*∗*^
P6	129.72 ± 64.22	125.2 (51.8–202.2)	0.647^*∗*^	0.618^*∗*^
P7	165.96 ± 60.80	180.8 (99–244.6)	0.579^*∗*^	0.466^*∗*^
P8	143.80 ± 46.77	141.6 (78.2–208.6)	0.849^*∗*^	0.593^*∗*^
P9	155.08 ± 40.78	169.2 (97–204.8)	0.813^*∗*^	0.590^*∗*^
P10	222.04 ± 45.61	242.2 (168.8–265)	0.144^*∗*^	0.120^*∗*^

*Note.* ^*∗*^Normal (*p* > 0.05).

**Table 3:** Differences in fibroblasts assessed using the Kruskal–Wallis test.

Group	Median (min-max)	*p*
P1	69.2 (53–108.2)	<0.001^*∗*^
P2	64 (54.6–99.4)	
P3	106.6 (57–117)	
P4	110.6 (38–116.8)	
P5	110.6 (39.2–157.2)	
P6	125.2 (51.8–202.2)	
P7	180.8 (99–244.6)	
P8	141.6 (78.2–208.6)	
P9	169.2 (97–204.8)	
P10	242.2 (168.8–265)	

**Table 4:** The result of the Mann–Whitney test for testing differences.

Group	P1	P2	P3	P4	P5	P6	P7	P8	P9	P10
P1	—	0.602	0.175	0.117	0.465	0.251	0.028^*∗*^	0.028^*∗*^	0.028^*∗*^	0.009^*∗*^
P2		—	0.047^*∗*^	0.117	0.347	0.175	0.016^*∗*^	0.028^*∗*^	0.016^*∗*^	0.009^*∗*^
P3			—	0.465	0.602	0.465	0.117	0.076	0.076	0.009^*∗*^
P4				—	0.675	0.347	0.209	0.076	0.076	0.009^*∗*^
P5					—	0.347	0.142	0.175	0.076	0.009^*∗*^
P6						—	0.465	0.602	0.465	0.076
P7							—	0.602	0.602	0.251
P8								—	0.602	0.028^*∗*^
P9									—	0.076

*Note.* ^*∗*^Significant (*p* < 0.05).

**Table 5:** Descriptive statistics and normality testing of collagen thickness data using the Shapiro–Wilk test in micrometer.

Group	Mean ± SD	Median (min-max)	*p*	Transf.
P1	1036.67 ± 277.16	986.87 (810.85–1486.59)	0.228^*∗*^	0.372^*∗*^
P2	995.20 ± 147.83	902.67 (867.01–1157.06)	0.030	0.036
P3	2011.76 ± 2480.2	944.31 (763.23–6446)	0.000	0.003
P4	787.20 ± 234.24	863.39 (427.39–1007.3)	0.498^*∗*^	0.237^*∗*^
P5	890.25 ± 240.61	1005.69 (560.25–1092.73)	0.188^*∗*^	0.164^*∗*^
P6	1223.88 ± 103.52	1274.16 (1042.92–1292.34)	0.018	0.013
P7	784.80 ± 185.51	739 (623.48–1105.01)	0.059^*∗*^	0.149^*∗*^
P8	942.15 ± 240.23	949.92 (594.83–1262.55)	0.869^*∗*^	0.632^*∗*^
P9	1013.51 ± 70.88	1013.29 (941.92–1113.89)	0.639^*∗*^	0.655^*∗*^
P10	992.11 ± 253.34	1023.47 (658.38–1324.91)	0.992^*∗*^	0.935^*∗*^

*Note.* ^*∗*^Normal (*p* > 0.05).

**Table 6:** The differences in collagen thickness in micrometer assessed using the Kruskal–Wallis test.

Group	Median (min-max)	*p*
P1	986.87 (810.85–1486.59)	0.115
P2	902.67 (867.01–1157.06)	
P3	944.31 (763.23–6446)	
P4	863.39 (427.39–1007.3)	
P5	1005.69 (560.25–1092.73)	
P6	1274.16 (1042.92–1292.34)	
P7	739 (623.48–1105.01)	
P8	949.92 (594.83–1262.55)	
P9	1013.29 (941.92–1113.89)	
P10	1023.47 (658.38–1324.91)	

*Note.* ^*∗*^Significant (*p* < 0.05).

## Data Availability

The Excel data used to support the findings of this study are available from the corresponding author upon request.
